# Supplementary material for: Craniofacial shape transition across the house mouse hybrid zone: implications for the genetic architecture and evolution of between-species differences
Source: Dev Genes Evol. 2016 May 23;226:173–86. doi: 10.1007/s00427-016-0550-7 (PMC4896993; doi:10.1007/s00427-016-0550-7)
Supplement: Supplementary file 1 — (DOCX 66 kb) [file 427_2016_550_MOESM1_ESM.docx]

## Supplementary Tables

**Supplementary Table S1. Percentage of *M. m. domesticus* alleles per individual.** The percentage of *M. m. domesticus* alleles (PercDom) based on the average of parental genotypes for 37 diagnostic SNPs is shown for all individuals used in this study. ID, identification number of the respective mouse (corresponds to MID in SNP genotype data (dryad cite). A, mouse was previously used in [Pallares *et al.* (2014](#_ENREF_38)) and [Turner and Harr (2014](#_ENREF_51)). These mice were genotyped with the panel of 270 SNPs. B, mouse used only in the current study.

| ID | Family ID | PercDom | Age (days) | Sex | OtherStudies |
| --- | --- | --- | --- | --- | --- |
| 1 | TUP9 | 21.7 | 73 | M | A |
| 2 | FSP14 | 59.2 | 80 | M | A |
| 3 | GLP6 | 14.6 | 64 | M | B |
| 4 | FSP14 | 59.2 | 80 | M | A |
| 5 | RFP2 | 15.0 | 81 | M | A |
| 6 | HAP9 | 25.0 | 84 | M | A |
| 7 | HAP8 | 24.2 | 83 | M | A |
| 8 | KHP1 | 22.9 | 84 | M | A |
| 9 | REP1 | 14.2 | 83 | M | A |
| 10 | GRP1 | 8.3 | 79 | M | B |
| 11 | GLP4 | 14.3 | 83 | M | A |
| 12 | HHP1 | 44.2 | 86 | M | A |
| 13 | KTP3 | 24.6 | 79 | M | A |
| 14 | STP1 | 93.3 | 83 | M | A |
| 15 | HAP7 | 27.5 | 81 | M | A |
| 16 | TUP5 | 20.8 | 81 | M | A |
| 17 | FSP10 | 60.8 | 72 | M | A |
| 18 | KTP1 | 27.2 | 79 | M | A |
| 19 | FSP10 | 60.8 | 75 | M | A |
| 20 | GRP1 | 8.3 | 69 | M | B |
| 21 | FHP2 | 37.5 | 76 | M | A |
| 22 | FTP1 | 42.5 | 84 | M | A |
| 24 | TUP9 | 21.7 | 73 | M | A |
| 25 | KTP1 | 27.2 | 82 | M | A |
| 26 | HHP1 | 44.2 | 86 | M | A |
| 27 | SOP7 | 88.8 | 84 | M | B |
| 28 | FSP7 | 61.0 | 81 | M | A |
| 30 | HAP4 | 23.3 | 76 | M | A |
| 31 | REP1 | 14.2 | 81 | M | B |
| 32 | KMP4 | 26.7 | 82 | M | A |
| 33 | FHP2 | 37.5 | 81 | M | A |
| 34 | SOP4 | 99.3 | 81 | M | B |
| 35 | REP1 | 14.2 | 83 | M | B |
| 36 | TSP1 | 60.0 | 84 | M | A |
| 37 | KTP3 | 24.6 | 79 | M | A |
| 38 | FSP12 | 56.8 | 71 | M | A |
| 39 | FSP12 | 56.8 | 68 | M | A |
| 40 | STP2 | 90.8 | 83 | M | A |
| 41 | KFP1 | 43.9 | 69 | M | A |
| 42 | HAP6 | 23.3 | 64 | M | A |
| 43 | KHP1 | 22.9 | 84 | M | A |
| 44 | HAP6 | 23.3 | 62 | M | A |
| 45 | HAP5 | 30.8 | 84 | M | A |
| 46 | RTP1 | 16.7 | 79 | M | A |
| 48 | HAP4 | 23.3 | 84 | M | A |
| 49 | FHP1 | 40.0 | 82 | M | A |
| 50 | FHP2 | 37.5 | 79 | M | A |
| 51 | KTP3 | 24.6 | 80 | M | A |
| 52 | SOP6 | 91.4 | 81 | M | A |
| 53 | KMP4 | 26.7 | 82 | M | A |
| 54 | KMP4 | 26.7 | 82 | M | A |
| 55 | TUP6 | 32.3 | 69 | M | A |
| 56 | STP6 | 88.3 | 64 | M | B |
| 57 | STP1 | 93.3 | 84 | M | B |
| 58 | HAP5 | 30.8 | 84 | M | A |
| 59 | HAP1 | 25.0 | 84 | M | A |
| 60 | KFP1 | 43.9 | 69 | M | A |
| 61 | FHP1 | 40.0 | 82 | M | A |
| 64 | FHP1 | 40.0 | 82 | M | A |
| 65 | KTP3 | 24.6 | 79 | M | A |
| 66 | KMP2 | 32.0 | 81 | M | A |
| 67 | FSP11 | 61.7 | 68 | M | A |
| 68 | KHP1 | 22.9 | 84 | M | A |
| 69 | HAP4 | 23.3 | 77 | M | A |
| 70 | KHP2 | 30.5 | 82 | M | A |
| 71 | HAP9 | 25.0 | 84 | M | A |
| 72 | KHP3 | 25.8 | 72 | M | A |
| 73 | REP1 | 14.2 | 82 | M | B |
| 74 | RTP1 | 16.7 | 81 | M | A |
| 75 | KTP3 | 24.6 | 79 | M | A |
| 76 | TUP5 | 20.8 | 76 | M | A |
| 77 | TUP2 | 29.7 | 84 | M | A |
| 78 | SOP5 | 91.1 | 76 | M | A |
| 80 | FSP8 | 60.0 | 81 | M | A |
| 81 | TUP5 | 20.8 | 81 | M | A |
| 82 | TSP2 | 61.4 | 84 | M | A |
| 84 | FHP2 | 37.5 | 80 | M | A |
| 85 | HOP5 | 69.4 | 67 | M | A |
| 86 | FTP1 | 42.5 | 84 | M | A |
| 87 | TSP2 | 61.4 | 84 | M | A |
| 88 | TUP3 | 18.3 | 77 | M | A |
| 89 | FSP4 | 56.7 | 84 | M | A |
| 90 | FTP2 | 34.2 | 75 | M | A |
| 91 | HAP1 | 25.0 | 83 | M | A |
| 92 | HAP3 | 17.5 | 83 | M | A |
| 93 | KTP1 | 27.2 | 82 | M | A |
| 94 | HAP9 | 25.0 | 84 | M | A |
| 95 | HHP1 | 44.2 | 86 | M | A |
| 96 | FSP11 | 61.7 | 71 | M | A |
| 97 | GLP2 | 15.8 | 83 | M | B |
| 98 | FTP2 | 34.2 | 75 | M | A |
| 99 | HAP1 | 25.0 | 82 | M | A |
| 100 | FSP12 | 56.8 | 68 | M | A |
| 101 | KMP1 | 22.4 | 81 | M | A |
| 102 | HHP1 | 44.2 | 86 | M | A |
| 103 | FHP1 | 40.0 | 81 | M | A |
| 104 | SGP1 | 50.3 | 80 | M | A |
| 106 | HAP4 | 23.3 | 77 | M | A |
| 107 | HOP4 | 69.4 | 84 | M | A |
| 108 | KTP1 | 27.2 | 82 | M | A |
| 109 | HOP4 | 69.4 | 84 | M | A |
| 110 | KMP1 | 22.4 | 82 | M | A |
| 111 | GLP6 | 14.6 | 62 | M | A |
| 112 | FSP6 | 55.8 | 82 | M | A |
| 113 | FSP8 | 60.0 | 78 | M | A |
| 114 | FSP3 | 55.0 | 84 | M | A |
| 115 | HAP6 | 23.3 | 69 | M | A |
| 116 | HAP3 | 17.5 | 83 | M | A |
| 117 | KHP1 | 22.9 | 83 | M | A |
| 118 | REP1 | 14.2 | 83 | M | B |
| 119 | KFP1 | 43.9 | 79 | M | A |
| 120 | KTP1 | 27.2 | 81 | M | A |
| 121 | FSP12 | 56.8 | 69 | M | A |
| 123 | TUP3 | 18.3 | 77 | M | A |
| 124 | FHP2 | 37.5 | 79 | M | A |
| 125 | TUP5 | 20.8 | 76 | M | A |
| 126 | HAP6 | 23.3 | 69 | M | A |
| 127 | RTP1 | 16.7 | 83 | M | A |
| 128 | KMP2 | 32.0 | 80 | M | A |
| 129 | HOP5 | 69.4 | 67 | M | A |
| 130 | TSP2 | 61.4 | 84 | M | A |
| 131 | HAP4 | 23.3 | 84 | M | A |
| 132 | FHP1 | 40.0 | 80 | M | A |
| 133 | HAP3 | 17.5 | 84 | M | A |
| 134 | SOP6 | 91.4 | 81 | M | B |
| 135 | TUP4 | 24.7 | 84 | M | A |
| 136 | HAP3 | 17.5 | 84 | M | A |
| 137 | FHP2 | 37.5 | 79 | M | A |
| 138 | TUP5 | 20.8 | 77 | M | A |
| 140 | HAP9 | 25.0 | 83 | M | A |
| 141 | KHP3 | 25.8 | 71 | M | A |
| 142 | TUP5 | 20.8 | 81 | M | A |
| 143 | SOP1 | 90.5 | 84 | M | B |
| 144 | TSP2 | 61.4 | 84 | M | A |
| 145 | TUP1 | 39.7 | 84 | M | A |
| 146 | HAP3 | 17.5 | 84 | M | A |
| 147 | TUP3 | 18.3 | 83 | M | A |
| 148 | SOP1 | 90.5 | 84 | M | B |
| 149 | KFP1 | 43.9 | 80 | M | A |
| 150 | TUP3 | 18.3 | 82 | M | A |
| 151 | KTP1 | 27.2 | 82 | M | A |
| 152 | FSP5 | 58.5 | 83 | M | A |
| 153 | KTP1 | 27.2 | 82 | M | A |
| 154 | TSP1 | 60.0 | 84 | M | A |
| 156 | HAP4 | 23.3 | 84 | M | A |
| 158 | HTP2 | 49.2 | 83 | M | A |
| 159 | GLP4 | 14.3 | 83 | M | B |
| 160 | HAP4 | 23.3 | 83 | M | A |
| 161 | TUP5 | 20.8 | 76 | M | A |
| 162 | TUP3 | 18.3 | 83 | M | A |
| 163 | TUP5 | 20.8 | 76 | M | A |
| 164 | RTP1 | 16.7 | 83 | M | A |
| 165 | RGP1 | 11.7 | 69 | M | B |
| 166 | KHP1 | 22.9 | 83 | M | A |
| 167 | FHP2 | 37.5 | 79 | M | A |
| 168 | RGP1 | 11.7 | 69 | M | B |
| 169 | KHP2 | 30.5 | 82 | M | A |
| 170 | RTP1 | 16.7 | 84 | M | A |
| 171 | FSP6 | 55.8 | 82 | M | A |
| 172 | HAP5 | 30.8 | 84 | M | A |
| 173 | HOP4 | 69.4 | 84 | M | A |
| 174 | FTP2 | 34.2 | 75 | M | A |
| 175 | GSP1 | 57.0 | 80 | M | A |
| 176 | KTP1 | 27.2 | 82 | M | A |
| 177 | KHP2 | 30.5 | 82 | M | A |
| 178 | STP4 | 90.0 | 81 | M | B |
| 180 | SOP7 | 88.8 | 84 | M | A |
| 181 | RTP1 | 16.7 | 84 | M | A |
| 182 | KTP3 | 24.6 | 80 | M | A |
| 184 | FTP1 | 42.5 | 84 | M | A |
| 185 | GLP2 | 15.8 | 83 | M | A |
| 186 | HAP6 | 23.3 | 64 | M | A |
| 187 | SOP1 | 90.5 | 84 | M | B |
| 188 | KHP3 | 25.8 | 71 | M | A |
| 190 | FHP2 | 37.5 | 83 | M | A |
| 191 | KHP2 | 30.5 | 83 | M | A |
| 192 | FSP8 | 60.0 | 82 | M | A |
| 193 | FSP13 | 57.5 | 83 | M | A |
| 194 | KHP3 | 25.8 | 71 | M | A |
| 195 | KHP3 | 25.8 | 72 | M | A |
| 196 | FSP10 | 60.8 | 72 | M | A |
| 197 | FSP5 | 58.5 | 83 | M | A |
| 198 | TUP5 | 20.8 | 82 | M | A |
| 199 | KTP1 | 27.2 | 82 | M | A |
| 200 | KHP2 | 30.5 | 83 | M | A |
| 211 | FSP7 | 61.0 | 71 | M | A |
| 214 | FSP7 | 61.0 | 71 | M | A |
| 215 | FSP17 | 59.2 | 73 | M | A |
| 222 | FSP14 | 59.2 | 71 | M | A |
| 225 | FSP16 | 51.7 | 71 | M | A |
| 230 | KMP3 | 24.0 | 74 | M | A |
| 233 | FSP7 | 61.0 | 71 | M | A |
| 236 | HFP3 | 60.8 | 84 | M | A |
| 240 | FSP16 | 51.7 | 72 | M | A |
| 244 | HOP11 | 66.1 | 74 | M | A |
| 245 | FSP16 | 51.7 | 72 | M | A |
| 249 | KMP4 | 26.7 | 83 | M | A |
| 250 | KMP3 | 24.0 | 74 | M | A |
| 500 | GRP1 | 8.3 | 87 | F | B |
| 501 | GRP1 | 8.3 | 87 | F | B |
| 502 | GRP1 | 8.3 | 90 | F | B |
| 503 | GRP1 | 8.3 | 90 | F | B |
| 504 | RGP1 | 11.7 | 64 | F | B |
| 505 | RGP1 | 11.7 | 64 | F | B |
| 506 | RGP1 | 11.7 | 64 | F | B |
| 508 | GLP4 | 14.3 | 85 | F | B |
| 509 | GLP6 | 14.6 | 63 | F | B |
| 510 | GLP6 | 14.6 | 92 | F | B |
| 511 | GLP2 | 15.8 | 72 | F | B |
| 512 | GLP2 | 15.8 | 72 | F | B |
| 513 | GLP2 | 15.8 | 72 | F | B |
| 516 | FHP1 | 40.0 | 75 | F | B |
| 517 | FHP1 | 40.0 | 75 | F | B |
| 518 | FHP1 | 40.0 | 84 | F | B |
| 519 | FHP1 | 40.0 | 84 | F | B |
| 520 | FHP1 | 40.0 | 84 | F | B |
| 521 | FHP1 | 40.0 | 84 | F | B |
| 522 | KFP1 | 43.9 | 92 | F | B |
| 523 | KFP1 | 43.9 | 92 | F | B |
| 524 | KFP1 | 43.9 | 92 | F | B |
| 525 | KFP1 | 43.9 | 92 | F | B |
| 526 | KFP1 | 43.9 | 92 | F | B |
| 527 | HTP2 | 49.2 | 63 | F | B |
| 528 | HTP2 | 49.2 | 63 | F | B |
| 529 | HTP2 | 49.2 | 63 | F | B |
| 530 | HHP3 | 50.5 | 80 | F | B |
| 531 | HHP3 | 50.5 | 80 | F | B |
| 532 | HHP3 | 50.5 | 80 | F | B |
| 533 | HHP3 | 50.5 | 80 | F | B |
| 534 | HHP3 | 50.5 | 80 | F | B |
| 535 | FSP14 | 59.2 | 66 | F | B |
| 537 | TSP1 | 60.0 | 71 | F | B |
| 538 | TSP1 | 60.0 | 71 | F | B |
| 539 | FSP8 | 60.0 | 76 | F | B |
| 540 | FSP8 | 60.0 | 76 | F | B |
| 541 | FSP10 | 60.8 | 91 | F | B |
| 548 | FSP11 | 61.7 | 85 | F | B |
| 549 | STP5 | 89.8 | 93 | F | B |
| 550 | STP5 | 89.8 | 93 | F | B |
| 551 | STP5 | 89.8 | 93 | F | B |
| 552 | STP5 | 89.8 | 93 | F | B |
| 553 | STP5 | 89.8 | 93 | F | B |
| 554 | STP5 | 89.8 | 93 | F | B |
| 556 | SOP6 | 91.4 | 88 | F | B |
| 557 | SOP6 | 91.4 | 88 | F | B |
| 558 | SOP6 | 91.4 | 88 | F | B |
| 559 | SOP6 | 91.4 | 88 | F | B |
| 560 | SOP6 | 91.4 | 88 | F | B |
| 561 | SOP6 | 91.4 | 88 | F | B |

**Supplementary Table S2.** Description of the landmarks used for geometric morphometrics analyses.

| Landmark | Description |
| --- | --- |
| Skull |  |
| 1 | Nasal bones most anterior suture |
| 2 | Nasal bones most posterior suture |
| 3 | Frontal bones most posterior suture |
| 4 | Parietal bones most posterior suture |
| 5 | Interparietal bone most posterior point on the median line |
| 6 | Right side, most anterior point of the suture between frontal and parietal bones |
| 7 | Left side, most anterior point of the suture between frontal and parietal bones |
| 8 | Right side, intersection between parietal, occipital and squamosal bones |
| 9 | Left side, intersection between parietal, occipital and squamosal bones |
| 10 | Right, most posterior junction of squamosal bone and the zygomatic process of the squamosal bone |
| 11 | Left, most posterior junction of squamosal bone and the zygomatic process of the squamosal bone |
| 12 | Right side, most anterior suture of the zygomatic process of the squamosal bone and jugal bone |
| 13 | Left side, most anterior suture of the zygomatic process of the squamosal bone and jugal bone |
| 14 | Right side, most anterior suture of jugal bone and the zygomatic process of the maxillary bone |
| 15 | Left side, most anterior suture of jugal bone and the zygomatic process of the maxillary bone |
| 16 | Right side, intersection of the frontal, lacrimal and the zygomatic process of the maxillary bone |
| 17 | Left side, intersection of the frontal, lacrimal and the zygomatic process of the maxillary bone |
| 18 | Right infraorbital foramen most superior point |
| 19 | Left infraorbital foramen most superior point |
| 20 | Right infraorbital foramen most inferior point |
| 21 | Left infraorbital foramen most inferior point |
| 22 | Right premaxilla-right nasal bone most anterior point of suture |
| 23 | Left premaxilla-left nasal bone most anterior point of suture |
| 24 | Most superior point of the right incisor alveolus |
| 25 | Most superior edge of the left incisor alveolus |
| 26 | Most inferior point of the right incisor alveolus |
| 27 | Most inferior point of the left incisor alveolus |
| 28 | Right premaxilla-maxilla most ventral juntion |
| 29 | Left premaxilla-maxilla most ventral juntion |
| 30 | Most anterior point of the right first molar alveolus |
| 31 | Most anterior point of the left first molar alveolus |
| 32 | Most posterior point of the right third molar alveolus |
| 33 | Most posterior point of the left third molar alveolus |
| 34 | Most anterior point of the right anterior palatine foramen |
| 35 | Most anterior point of the left anterior palatine foramen |
| 36 | Most posterior point of the right anterior palatine foramen |
| 37 | Most posterior point of the left anterior palatine foramen |
| 38 | Right pterygoid process, most posterior point |
| 39 | Left pterygoid process, most posterior point |
| 40 | Median-line point of the suture between occipital and basisphenoid bones |
| 41 | Median-line point of the suture between basisphenoid and presphenoid bones |
| 42 | Most posterior point of the suture between palatine bones |
| 43 | Foramen magnum most anterior point, Basion |
| 44 | Foramen magnum most posterior point, Bregma |
|  |  |
| Mandible |  |
| 1 | Most superior point of the incisor alveolus |
| 2 | Most inferior point of the incisor alveolus |
| 3 | Most anterior point of the first molar alveolus |
| 4 | Most posterior point of the third molar alveolus |
| 5 | Most posterior tip of the coronoid process |
| 6 | Most anterior concave point of coronoid process |
| 7 | Most anterior point of the articular surface of the condyle |
| 8 | Most posterior tip of the condyle |
| 9 | Most anterior concave point between condyle and angular process |
| 10 | Most posterior tip of angular process |
| 11 | Most inferior point of angular process |
| 12 | Ascending ramus dorsal-most ventral point |
| 13 | Alveolar region most inferior point |

**Supplementary Table S3. Results of comparisons between the skull shapes of hybrid groups.** VarPCs, amount of skull variation represented by the first 10 PCs from a PCA using individuals from both groups. DiffMean, p-value after multiple testing correction from a MANOVA using the first 10 PCs. MisClass, percentage of mice classified to the wrong group in a leave-one-out validation of the discriminant analysis. Procrustes, Procrustes distance between mean skull shapes. Genomic, difference between the mean percentage of *M. m. domesticus* alleles. Angle, angle between *mus*-to-*dom* vector and the vector formed by the mean shape of the groups. SignAngle, p-value after multiple testing correction of the angle between vectors.

| GroupPair | VarPCs | DiffMean | Misclass | Procrustes | Genomic | Angle | SignAngle |
| --- | --- | --- | --- | --- | --- | --- | --- |
| 0-1 | 0.81162 | 4.00E-05 | 8.9 | 0.0266 | 7.2 | 90.3 | 0.88068 |
| 0-2 | 0.76457 | 3.37E-09 | 1.4 | 0.0254 | 15.9 | 87.2 | 0.88068 |
| 0-3 | 0.89436 | 2.25E-06 | 0 | 0.0298 | 25.9 | 78.8 | 0.282 |
| 0-4 | 0.84081 | 2.94E-09 | 0 | 0.0295 | 34.8 | 65.9 | 0.00351 |
| 0-5 | 0.85707 | 5.31E-06 | 6.2 | 0.0319 | 46.9 | 61.8 | 0.00036 |
| 0-6 | 0.83864 | 6.46E-10 | 0 | 0.0320 | 54.0 | 60.3 | 0.00036 |
| 0-8 | 0.9653 | 6.94E-08 | 6.7 | 0.0326 | 81.1 | 44.4 | 0.00036 |
| 0-9 | 0.90405 | 2.94E-08 | 4.3 | 0.0378 | 83.5 | 43.3 | 0.00036 |
| 1-2 | 0.73235 | 1.50E-11 | 15 | 0.0140 | 8.7 | 84.2 | 0.82564 |
| 1-3 | 0.7898 | 3.27E-09 | 9.8 | 0.0182 | 18.7 | 71.0 | 0.04056 |
| 1-4 | 0.77688 | 5.03E-14 | 8.6 | 0.0231 | 27.6 | 58.1 | 0.00036 |
| 1-5 | 0.78354 | 4.45E-17 | 4.6 | 0.0264 | 39.7 | 54.8 | 0.00036 |
| 1-6 | 0.77005 | 3.86E-20 | 0 | 0.0240 | 46.8 | 48.2 | 0.00036 |
| 1-8 | 0.80186 | 2.97E-13 | 0 | 0.0275 | 73.9 | 31.5 | 0.00036 |
| 1-9 | 0.79815 | 9.17E-20 | 0 | 0.0284 | 76.3 | 13.6 | 0.00036 |
| 2-3 | 0.76008 | 5.01E-09 | 11 | 0.0149 | 9.9 | 72.3 | 0.05283 |
| 2-4 | 0.73954 | 5.07E-24 | 2.1 | 0.0171 | 18.9 | 50.9 | 0.00036 |
| 2-5 | 0.75632 | 3.78E-26 | 1.1 | 0.0249 | 31.0 | 56.3 | 0.00036 |
| 2-6 | 0.74595 | 8.86E-32 | 2.1 | 0.0231 | 38.1 | 50.8 | 0.00036 |
| 2-8 | 0.76545 | 9.17E-20 | 0 | 0.0287 | 65.2 | 39.9 | 0.00036 |
| 2-9 | 0.7726 | 5.76E-29 | 0 | 0.0301 | 67.6 | 29.3 | 0.00036 |
| 3-4 | 0.79658 | 2.40E-09 | 5.7 | 0.0140 | 8.9 | 63.3 | 9.00E-04 |
| 3-5 | 0.81162 | 2.79E-07 | 8.3 | 0.0167 | 21.1 | 56.1 | 0.00036 |
| 3-6 | 0.79394 | 3.19E-13 | 3.8 | 0.0185 | 28.2 | 57.0 | 0.00036 |
| 3-8 | 0.86897 | 2.29E-09 | 0 | 0.0281 | 55.3 | 51.4 | 0.00036 |
| 3-9 | 0.84593 | 1.25E-12 | 0 | 0.0281 | 57.6 | 39.6 | 0.00036 |
| 4-5 | 0.77301 | 2.25E-06 | 14 | 0.0129 | 12.1 | 76.4 | 0.18879 |
| 4-6 | 0.76598 | 4.93E-08 | 18 | 0.0140 | 19.2 | 74.2 | 0.10008 |
| 4-8 | 0.82494 | 5.79E-08 | 2.5 | 0.0253 | 46.3 | 63.6 | 0.00098 |
| 4-9 | 0.81042 | 4.98E-12 | 2.1 | 0.0252 | 48.7 | 52.2 | 0.00036 |
| 5-6 | 0.77073 | 1.20E-04 | 19 | 0.0116 | 7.1 | 86.2 | 0.88068 |
| 5-8 | 0.84327 | 5.90E-09 | 0 | 0.0257 | 34.2 | 71.4 | 0.04367 |
| 5-9 | 0.81915 | 1.06E-14 | 0 | 0.0251 | 36.6 | 60.4 | 0.00036 |
| 6-8 | 0.82006 | 1.03E-06 | 5 | 0.0232 | 27.1 | 71.4 | 0.04367 |
| 6-9 | 0.80188 | 2.79E-09 | 4.2 | 0.0217 | 29.5 | 57.6 | 0.00036 |
| 8-9 | 0.87154 | 0.2126444 | 27 | 0.0202 | 2.4 | 78.0 | 0.26676 |
| mus-dom | 0.79065 | 2.37E-28 | 0 | 0.0261 | 78.7 | - | - |

**Supplementary Table S4. Results of comparisons between the mandible shape of hybrid groups.** VarPCs, amount of skull variation represented by the first 10 PCs from a PCA using individuals from both groups. DiffMean, p-value after multiple testing correction from a MANOVA using the first 10 PCs. MisClass, percentage of mice classified to the wrong group in a leave-one-out validation of the discriminant analysis. Procrustes, Procrustes distance between mean skull shapes. Genomic, difference between the mean percentage of *M. m. domesticus* alleles. Angle, angle between *mus*-to-*dom* vector and the vector formed by the mean shape of the groups. SignAngle, p-value after multiple testing correction of the angle between vectors.

| GroupPair | VarPCs | DiffMean | Misclass | Procrustes | Genomic | Angle | SignAngle |
| --- | --- | --- | --- | --- | --- | --- | --- |
| 0-1 | 0.87664 | 0.0065 | 6.7 | 0.02618 | 7.2 | 89.1 | 1 |
| 0-2 | 0.85102 | 1.1E-06 | 1.4 | 0.02840 | 16.0 | 86.1 | 1 |
| 0-3 | 0.88724 | 9.4E-05 | 3.4 | 0.02702 | 25.7 | 74.8 | 0.4180 |
| 0-4 | 0.86911 | 1.8E-09 | 0 | 0.02642 | 34.7 | 58.5 | 1.3E-02 |
| 0-5 | 0.89918 | 8.9E-06 | 6.2 | 0.03228 | 46.9 | 63.0 | 4.7E-02 |
| 0-6 | 0.8741 | 4.3E-07 | 2.7 | 0.03662 | 54.0 | 54.5 | 3.4E-03 |
| 0-8 | 0.9737 | 5.3E-05 | 6.7 | 0.04751 | 81.1 | 33.4 | 3.6E-04 |
| 0-9 | 0.94324 | 2.1E-05 | 0 | 0.05052 | 83.5 | 28.2 | 3.6E-04 |
| 1-2 | 0.83109 | 4.5E-08 | 21 | 0.01217 | 8.8 | 82.9 | 0.9850 |
| 1-3 | 0.85401 | 4.0E-08 | 13 | 0.01716 | 18.6 | 67.0 | 0.1241 |
| 1-4 | 0.84424 | 9.8E-12 | 10 | 0.02191 | 27.6 | 52.3 | 1.6E-03 |
| 1-5 | 0.86367 | 8.5E-13 | 4.6 | 0.02552 | 39.7 | 56.0 | 6.1E-03 |
| 1-6 | 0.852 | 1.6E-14 | 2.9 | 0.02797 | 46.8 | 41.7 | 3.6E-04 |
| 1-8 | 0.88303 | 5.8E-15 | 2.1 | 0.04162 | 73.9 | 19.5 | 3.6E-04 |
| 1-9 | 0.8867 | 6.0E-16 | 0 | 0.04481 | 76.3 | 10.2 | 3.6E-04 |
| 2-3 | 0.83272 | 0.0002 | 23 | 0.01504 | 9.8 | 69.8 | 0.1728 |
| 2-4 | 0.83292 | 2.2E-14 | 11 | 0.02241 | 18.8 | 58.0 | 0.0120 |
| 2-5 | 0.84601 | 7.4E-14 | 13 | 0.02445 | 30.9 | 58.6 | 0.0127 |
| 2-6 | 0.83645 | 1.8E-20 | 4.1 | 0.02865 | 38.0 | 47.5 | 3.6E-04 |
| 2-8 | 0.85933 | 1.1E-16 | 1.3 | 0.04139 | 65.1 | 24.2 | 3.6E-04 |
| 2-9 | 0.86719 | 2.5E-23 | 0 | 0.04522 | 67.5 | 19.6 | 3.6E-04 |
| 3-4 | 0.84507 | 0.0002 | 19 | 0.01782 | 9.0 | 67.9 | 0.1337 |
| 3-5 | 0.86057 | 0.0001 | 16 | 0.01998 | 21.2 | 67.7 | 0.1337 |
| 3-6 | 0.84548 | 1.4E-07 | 11 | 0.02331 | 28.3 | 52.5 | 1.7E-03 |
| 3-8 | 0.89106 | 4.0E-08 | 0 | 0.03824 | 55.3 | 31.7 | 3.6E-04 |
| 3-9 | 0.88634 | 3.7E-11 | 0 | 0.04100 | 57.8 | 24.1 | 3.6E-04 |
| 4-5 | 0.85158 | 0.03778 | 33 | 0.01332 | 12.1 | 86.2 | 1 |
| 4-6 | 0.83065 | 6.3E-06 | 13 | 0.01722 | 19.2 | 64.2 | 0.0631 |
| 4-8 | 0.8826 | 1.8E-09 | 0 | 0.03425 | 46.3 | 41.0 | 3.6E-04 |
| 4-9 | 0.87883 | 1.7E-14 | 0 | 0.03468 | 48.7 | 27.6 | 3.6E-04 |
| 5-6 | 0.8325 | 0.0248 | 28 | 0.01256 | 7.1 | 58.2 | 0.0125 |
| 5-8 | 0.89743 | 1.2E-07 | 0 | 0.03429 | 34.2 | 43.2 | 3.6E-04 |
| 5-9 | 0.89319 | 5.3E-11 | 0 | 0.03723 | 36.6 | 36.7 | 3.6E-04 |
| 6-8 | 0.87383 | 1.2E-07 | 2.5 | 0.02852 | 27.1 | 49.9 | 6.3E-04 |
| 6-9 | 0.86648 | 2.6E-08 | 6.2 | 0.03086 | 29.5 | 41.2 | 3.6E-04 |
| 8-9 | 0.92581 | 0.0690 | 35 | 0.02107 | 2.4 | 76.7 | 0.4904 |
| mus-dom | 0.87326 | 2.0E-26 | 0 | 0.04239 | 78.7 | - | - |

**Supplementary Table S5 Mean shape comparisons between hybrid groups.** Skull and mandible results are shown. The lower triangle (dark green) shows the p-value from Hotelling T2-test after correcting for multiple testing. T2 statistics and degrees of freedom (in parenthesis) are shown. The upper triangle (light green) shows the percentage of misclassified individuals derived from a linear discriminant analysis using a leave-one-out cross-validation procedure. In red are the pairwise comparisons for groups with similar mean shape.

| SKULL | 0 | 1 | 2 | 3 | 4 | 5 | 6 | 8 | 9 |
| --- | --- | --- | --- | --- | --- | --- | --- | --- | --- |
| 0 |  | 9% | 1% | 0% | 0% | 6% | 0% | 7% | 4% |
| 1 | T^2^(10,34) = 6.6  P = 4E-05 |  | 15% | 10% | 9% | 5% | 0% | 0% | 0% |
| 2 | T^2^(10,60) = 11  P = 3E-09 | T^2^(10,93) = 11.8  P = 2E-11 |  | 11% | 2% | 1% | 2% | 0% | 0% |
| 3 | T^2^(10,17) = 18  P = 2E-06 | T^2^(10,50) = 12.3  P = 3E-09 | T^2^(10,76) = 9.6  P = 5E-09 |  | 6% | 8% | 4% | 0% | 0% |
| 4 | T^2^(10,26) = 23.2  P = 3E-09 | T^2^(10,59) = 19.9  P = 5E-14 | T^2^(10,85) = 34  P = 5E-24 | T^2^(10,42) = 14.4  P = 2E-09 |  | 14% | 18% | 3% | 2% |
| 5 | T^2^(10,21) = 12  P = 5E-06 | T^2^(10, 54) = 30  P = 4E-17 | T^2^(10,80) = 42  P = 4E-26 | T^2^(10,37) = 10.6  P = 3E-07 | T^2^(10,46) = 7.8  P = 2E-06 |  | 19% | 0% | 0% |
| 6 | T^2^(10,26) = 27  P = 6E-10 | T^2^(10,59) = 36.6  P = 4E-20 | T^2^(10,85) = 56.7  P = 9E-32 | T^2^(10,42) = 25.2  P = 3E-13 | T^2^(10,51) = 10.1  P = 5E-08 | T^2^(10,46) = 5.1  P = 1E-04 |  | 5% | 4% |
| 8 | T^2^(10,4) = 76.5  P = 7E-08 | T^2^(10,37) = 26.6  P = 3E-13 | T^2^(10,63) = 32.8  P = 9E-20 | T^2^(10,20) = 36.6  P = 2E-09 | T^2^(10,29) = 15.1  P = 6E-08 | T^2^(10,24) = 23.7  P = 6E-09 | T^2^(10,29) = 11.3  P = 1E-06 |  | 27% |
| 9 | T^2^(10,12) = 77.8  P = 3E-08 | T^2^(10,45) = 51.2  P = 9E-20 | T^2^(10,71) = 59.6  P = 6E-29 | T^2^(10,28) = 40.6  P = 1E-12 | T^2^(10,37) = 24.6  P = 5E-12 | T^2^(10,32) = 46  P = 1E-14 | T^2^(10,37) = 15.8  P = 3E-09 | T^2^(10,15) = 1.6  P = **0.21** |  |
| MANDIBLE | 0 | 1 | 2 | 3 | 4 | 5 | 6 | 8 | 9 |
| 0 |  | 7% | 1% | 3% | 0% | 6% | 3% | 7% | 0% |
| 1 | T^2^(10,34) = 3.8  P = 6E-03 |  | 21% | 13% | 10% | 5% | 3% | 2% | 0% |
| 2 | T^2^(10,62) = 7.6  P = 1E-06 | T^2^(10,95) = 8.1  P = 5E-08 |  | 23% | 11% | 13% | 4% | 1% | 0% |
| 3 | T^2^(10,18) = 10.6  P = 9E-05 | T^2^(10,51) = 10.6  P = 4E-08 | T^2^(10,79) = 4.8  P = 2E-04 |  | 19% | 16% | 11% | 0% | 0% |
| 4 | T^2^(10,26) = 24.8  P = 2E-09 | T^2^(10,59) = 15.5  P = 1E-11 | T^2^(10,87) = 16  P = 2E-14 | T^2^(10,43) = 5.5  P = 2E-04 |  | 33% | 13% | 0% | 0% |
| 5 | T^2^(10,21) = 12.7  P = 9E-06 | T^2^(10,54) = 18.8  P = 9E-13 | T^2^(10,82) = 15.7  P = 7E-14 | T^2^(10,38) = 6.1  P = 1E-04 | T^2^(10,46) = 2.5  P = 0.038 |  | 28% | 0% | 0% |
| 6 | T^2^(10,26) = 14.5  P = 4E-07 | T^2^(10,59) = 21.2  P = 2E-14 | T^2^(10,87) = 25.9  P = 2E-20 | T^2^(10,43) = 10.6  P = 1E-07 | T^2^(10,51) = 7.3  P = 6E-06 | T^2^(10,46) = 2.8  P = 0.025 |  | 3% | 6% |
| 8 | T^2^(10,4) = 15.2  P = 9E-03 | T^2^(10,37) = 38.2  P = 6E-15 | T^2^(10,65) = 24.2  P = 1E-16 | T^2^(10,21) = 24.7  P = 4E-08 | T^2^(10,29) = 21.6  P = 2E-09 | T^2^(10,24) = 18  P = 1E-07 | T^2^(10,29) = 18.4  P = 1E-07 |  | 35% |
| 9 | T^2^(10,12) = 24  P = 2E-05 | T^2^(10,45) = 33  P = 6E-16 | T^2^(10,73) = 37.7  P = 3E-23 | T^2^(10,29) = 29.6  P = 4E-11 | T^2^(10,37) = 35.6  P = 2E-14 | T^2^(10,32) = 25  P = 5E-11 | T^2^(10,37) = 13.6  P = 3E-08 | T^2^(10,15) = 2.3  P = **0.069** |  |
